# Examining the relationship between health literacy and quality of life: evidence from older people admitted to the hospital

**DOI:** 10.1186/s12877-023-03838-w

**Published:** 2023-03-17

**Authors:** Gholamhossein Mehralian, Ali Reza Yusefi, Esmat Rezabeigi Davarani, Sudabeh Ahmadidarrehsima, Parnian Nikmanesh

**Affiliations:** 1 Nottingham Business School, Nottingham, UK; 2 Department of Public Health, School of Health, Jiroft University of Medical Sciences, Jiroft, Iran; 3 Health in Disasters and Emergencies Research Center, Institute for Future Studies in Health, Kerman University of Medical Sciences, Kerman, Iran; 4 Department of Midwifery, Nursing and Midwifery School, Jiroft University of Medical Sciences, Jiroft, Iran; 5 Healthcare Services Management, School of Management and Information Sciences, Iran University of Medical Sciences, Tehran, Iran; 6 School of Health, Jiroft University of Medical Sciences, Jiroft, Iran

**Keywords:** Health literacy, Quality of life, Older people, Hospital, Iran

## Abstract

**Introduction:**

Literacy has become an increasingly serious problem, especially as it relates to health care. In this regard, health literacy (HL), as a cognitive skill, has proven to be an influential factor to improve of the quality of life (QOL). This study aimed to examine the level of HL and its relationship with the QOL of older people at the time of discharge from the hospital in the south of Iran.

**Methods:**

This descriptive-analytical cross-sectional study included 300 older people admitted and treated in 10 teaching-therapeutic hospitals affiliated with the Shiraz University of Medical Sciences in 2021. The standard Health Literacy for Iranian Adults (HELIA) questionnaire and the World Health Organization Quality of Life Questionnaire (WHOQOL-BREF) were used to collect the required data. Data were analyzed with SPSS software version 23 software using descriptive and inferential statistics, Pearson’s correlation coefficient, T-test, ANOVA, and multiple linear regression at *p* = 0.05.

**Results:**

The mean scores of Hl and QOL for older people were 48.22 ± 9.63 (out of 100) and 61.59 ± 12.43 (out of 120), respectively. Moreover, there was a significant direct correlation between the participants’ HL and their QOL (*r*=0.388, *p*<0.001). All dimensions of HL, including comprehension (*β*=0.461, *p*<0.001), decision-making and behavior (*β*=0.434, *p*<0.001), access (*β*=0.397, *p*<0.001), reading skill (*β*=0.362, *p*=0.002), and assessment (*β*=0.278, *p*=0.004), were significant relationship with QOL. A statistically significant difference was revealed between the mean scores of HL regarding the participants’ gender (*p*=0.04) and level of education (*p*=0.001). Furthermore, the mean scores of QOL were significantly different with regard to older people’s gender (*p*=0.02), marital status (*p*=0.03), level of education (*p*=0.002), and income (*p*=0.01).

**Conclusion:**

The findings revealed the participants’ inadequate HL and average QOL. Considering the relationship of HL with QOL, it is recommended to develop comprehensive programs and effective interventions to develop HL skills and subsequently improve QOL among older people.

## Background

Aging is a sensitive phase of human life, and attention to the problems and needs of this age group is of paramount importance [1]. This phase of life is always considered one of the main economic, social, and health challenges in all countries [2]. According to estimates in 2012, older people account for 0.8% of the world’s population. Meanwhile, in 2015, the proportion of older people population increased by 0.5% and reached 8.5% of the total population 2015. The frequency of older people is predicted to reach one billion or 0.12 of the total population by 2030. By 2050, older people are expected to cover 16.7% of the total world population and reach above two billion persons [3]. On the other hand, older people in most societies are more likely to experience decreased physical, mental, and cognitive abilities and consequently hospitalization in health service centers such as hospitals. Accordingly, they are more likely to depend on formal and informal support to maintain health, performance, and self-sufficiency [4]. In this regard, attention to self-care and responsibility of older people towards different diseases is one of the support solutions requiring high levels of HL [5].

HL refers to individuals’ ability to interpret and understand basic information required for making decisions in the field of health [6]. This literacy encompasses skills, including reading, listening, analysis, and decision-making, and the ability to apply these skills in health situations, which are not necessarily correlated with years of education or general reading ability [7]. According to the World Health Organization, HL is defined not only as an individual character but also as a key determinant of health at the population level [8], which is a critical component in making appropriate and correct health decisions [9]. The appropriate level of HL makes individuals obey the orders of the health and treatment staff, promotes the effectiveness of medical consultations and health promotion and self-care programs, and enhances individuals’ desire to participate in screening programs [10]. On the other hand, inadequate and low HL in older people is associated with factors such as reduced cognitive ability, decreased physical health, increased risk of dementia, and risk factors of chronic diseases in this age group [11–13]. Moreover, some studies have documented that low HL in older people is associated with increased depression [14], adoption of some high-risk health behaviors [15], and generally unfavorable physical and mental health [16]. Accordingly, older people must reach an acceptable level of HL to maintain and improve their health level [17].

Moreover, experts believe that individuals with acceptable HL usually enjoy better QOL [18]. QOL is considered one of the key components of general health [19] and one of the indicators reflecting the health status of older people [20]. According to the WHO, QOL refers to individuals’ assessment and comprehension of their life situation under the influence of the cultural and value system and the setting where they live. Individuals’ goals, expectations, standards, and desires significantly affect their physical and psychological condition, level of independence, social relationships, and beliefs [21, 22]. Compared to other age groups, older people experience a unique QOL due to conditions such as older age and special experiences and skills [23]. On the other hand, changes in the disease models, i.e., a decrease in the rate of infectious diseases and an increase in life expectancy and chronic diseases, have led to increased attention to the concepts of health and QOL among older people in recent decades [24]. The importance of QOL is so much that experts have introduced the focus of health care in the current century to be developing QOL [25]. Examining the level of HL and QOL in older people can provide comprehensive information about their health conditions to promote their literacy level and consequently reach acceptable QOL in this age group. To this end, the present study aimed to examine the level of HL and its relationship with the QOL of older people at the time of discharge from the hospital settings.

## Hypothesis development

Physical impairment increases with age, and its negative effect on the ability to maintain independence increases the need for assistance, thereby decreasing QOL among older people [26]. In this regard, some studies have reported HL as one of the factors affecting QOL [27–32]. According to Wang et al. and Skevington et al., low HL is correlated with poor QOL, which can be caused by decreased accessibility and less use of medical care, poor self-management of the disease, reduced self-efficacy and ability to exercise control over life and surrounding environment, and increased stress aroused by daily life challenges [33, 34]. Yusefi et al. found a significant relationship between HL and QOL [11]. In their study, Panahi et al. reported a significant direct correlation between HL with the physical and mental dimensions of QOL and the overall QOL [35]. Kooshyar et al. also suggested that individuals with sufficient HL had a higher QOL and a significant relationship between HL and the physical and mental dimensions of QOL [28]. Zheng et al. [36], Couture et al. [29], and Wang et al. [33], also indicated that HL had an impact on QOL. Alnawajha claimed that QOL is the result of HL [37]. According to Hosieni et al., HL is the predictor of the QOL score [38]. Finally, Lee et al. state that HL in older people has a potential effect on promoting their health and QOL and decreases health care costs accordingly [39].

Moreover, some studies have revealed a significant relationship between HL and demographic variables such as gender [40–42] and level of education [40, 43–46]. Moreover, the relationship between QOL and variables such as gender [47–50], marital status [44, 50–52], level of education [52, 53], and income [48, 50] has also been documented in some other studies. According to the theoretical framework and various reviews of HL and QOL [11, 27–39], the following research hypotheses were formulated:

1. The HL and its dimensions are positively related to older people’s QOL.
2. The mean scores of HL and QOL for older people were significantly different regarding demographic variables.

## Methods

### Design and setting

This descriptive-analytical study was cross-sectional which conducted in first-level teaching therapeutic hospitals (n=10) affiliated with the Shiraz University of Medical Sciences in the south of Iran from July to November 2021.

### Participants

Participants in the study were older people who received medical advice in the discharge room of hospitals at the time of discharge. Most of these elderly people were in the internal medicine department of hospitals due to diseases such as gastrointestinal, cardiovascular, diabetes, and kidney problems.

According to the following Eq. [54] and given the correlation between HL and QOL (*r*=0.201) based on a pilot study in Iran [55], at a confidence level of 95% and *β*=0.1, the sample size was estimated to be at least 259 persons. To increase accuracy and avoid bias caused by sample size drop, 300 persons were included in the study.

1 $$n = {\left[ {\left( {{Z_1} - \alpha {/_2} + {\rm{ }}{Z_{1 - \beta }}} \right){\rm{ }}/{\rm{ }}W} \right]^2} + {\rm{ }}3$$

In Eq. (1), *W* is calculated using the following equation:

2 $$W = \raise.5ex\hbox{$\scriptstyle 1$}\kern-.1em/\kern-.15em\lower.25ex\hbox{$\scriptstyle 2$} {\rm{ }}ln\left( {1 + r/1 - r} \right)$$

In Eq. 2, *r* is the estimated coefficient of correlation between responsiveness and service quality in a pilot study in Iran [55]. Questionnaires were submitted to the 300 participants in hospitals, proportional to the frequency of older people population in each of the studied hospitals. For this purpose, after visiting each hospital, the total frequency of older people (> 60 years) at the time of discharge in different wards was recorded. After calculating the total number of older people, the proportional sampling method was used to distribute 300 questionnaires. Older people were randomly selected in each hospital according to the number of older people in each ward. Inclusion criteria were informed consent, willingness to participate in the study, 60 years of age or older, the ability to speak and communicate. According to the WHO, 60 years of age or older is defined as the cutoff point of old age in developing countries [56]. Since cognitive disorders such as delirium and major and minor neurocognitive disorders affect individuals’ cognitive abilities (learning, memory, perception, and concentration) [57], older people with these disorders were excluded from the study. To this end, with the help of the supervisor and the doctor, the patient’s medical records were reviewed, and those with the aforementioned disorders were detected. Additionally, people who did not wish to continue cooperating were excluded.

### Instruments

The data collection instrument was a three-section standard questionnaire. The first section of the questionnaire encompassed demographic information (including age, gender, marital status, level of education, and income), and the second and third sections contained the standard HELIA [58] and WHOQOL-BREF [59] questionnaires. The 33-item HELIA measures individuals’ ability in five subscales of health literacy, including reading skills (n=4), access (n=6), comprehension (n=7), assessment (n=4), and decision-making and behavior (n=12). The scale is scored on a 5-point Likert scale. The reading skill items are scored 5 (completely easy), 4 (easy), 3 (neither easy nor difficult), 2 (difficult), and 1 (completely difficult). For the other four subscales, the scoring scale contained 5 (always), 4 (often), 3 (sometimes), 2 (rarely), and 1 (never). To score the questionnaire, the raw scores in each subscale are obtained from the algebraic sum of the scores. Then, to convert this score into a range of zero to one hundred, the following formula is used: Adjusted score = (The raw score obtained-minimum possible score/maximum possible-minimum possible score). Finally, to obtain the total score, the scores of all dimensions (ranging from zero to one hundred) are added and divided by the number of dimensions (n=5). Then health literacy was categorized into four levels inadequate (0–50 points), not enough (50.1–66), adequate (66.1–84), and excellent (84.1–100) [11]. Montazeri et al.‘s confirmed the validity and reliability (Cronbach’s alpha coefficient=0.89) of this questionnaire [58].

The third section consisted of the standard WHOQOL-BREF questionnaire with 24 items in four domains: physical health (n=7 items), psychological health (n=6), social relations (n=3), and living environment (n=8). The items were scored on a 5-point Likert scale with 1 (very bad), 2 (bad), 3 (moderate), 4 (good), and 5 (very good). Older people’s QOL was also classified as follows: unfavorable (24–48), moderate (49–72), acceptable (73–96), and excellent (97–120). The validity and reliability of this questionnaire (with Cronbach’s alpha coefficient > 0.7) are confirmed in previous studies [59]. The validity and reliability of these two questionnaires are confirmed in previous studies [56, 59]. In the present study, the validity of the questionnaires was confirmed by 12 members of the academic staff and experts in the field of health and treatment management from the Iranian universities of medical sciences. Moreover, the calculated Content Validity Index (CVI) and Content Validity Ratio (CVR) were 0.88 and 0.90 for the HELIA questionnaire and 0.87 and 0.89 for the WHOQOL-BREF questionnaire, respectively. Regarding their reliability, a sample of 30 older people was pre-tested, and Cronbach’s alpha coefficient was 0.87 for the HELIA questionnaire and 0.79 for the WHOQOL-BREF questionnaire; hence, the reliability of the questionnaire was confirmed.

### Procedures and statistical analysis

To collect the required data, two researchers (PN and ERD) visited the hospitals on different weekdays in the morning, evening, and night shifts and distributed and collected questionnaires. To observe the ethical considerations, older people were voluntarily included in the study and filled out the questionnaires. After explaining the research objectives of the participants, they were ensured of the confidentiality of their information, and their verbal consent was obtained, and then the questionnaires were distributed in person among older people under study and collected on the same day. The questionnaire was completed by the participants. However, some older people asked the research team (PN and ERD) to help them complete the questionnaires. Then the collected data were imported to IBM SPSS software version 23. To investigate the correlation between older people’s HL and QOL and examine the correlation between these two variables and age, Pearson’s correlation coefficient was used. A T-test was used to detect the difference in the mean score of the two main research variables regarding gender. The ANOVA test was used to investigate the difference between the mean scores of HL and QOL in older people regarding their marital status, level of education, and income level. Finally, multiple linear regression was used to investigate the simultaneous relationship of different aspects of HL with QOL in older people.

## Results

The mean age of older people was 69.74 ± 5.23 years, with most of them (57.33%) being in the age group of 60–70 years. Moreover, 53.67% of the participants were females, and the others were males. Most of the respondents had elementary education (46%) and were married (85%) with an income of 10–20 million Rials (54.67%). Table 1 shows the frequency distribution of the participants’ demographic information.

**Table 1:** Frequency distribution of participants’ demographic information (n = 300)

Variables	Category	Frequency	%
**Age** **(year)**	60–70 71–80 80<	172 107 21	57.33 35.67 7
Total	------	300	100
**Gender**	Male Female	139 161	46.33 53.67
Total	------	300	100
**Marital Status**	Single Married Divorced Widow	7 255 9 29	2.33 85 3 9.67
Total	------	300	100
**Level of Education**	Unable to Read and Write Reading and Writing Elementary School Diploma BSc and higher	34 48 138 61 19	11.33 16 46 20.33 6.34
Total	------	300	100
**Income Level** **(Rials)**	< 10,000,000 10,000,000–20,000,000 20,000,001–30,000,000 > 30,000,000	73 164 49 14	24.33 54.67 16.33 4.67
Total	------	300	100

As presented in this table, the mean score of HL for older people was 48.22 ± 9.63 (out of 100), indicating inadequate HL. Among the HL dimensions, the maximum and minimum mean scores were obtained for access (50.19 ± 9.82 out of 100) and assessment (46.13 ± 9.32 out of 100). As presented in Table 2, the mean score of the participants’ QOL was 61.59 ± 12.43 out of 120, indicating their moderate QOL (Table 2).

**Table 2:** Mean and standard deviation of participants’ HL and QOL

Main Variable	Dimension	Score Domain	Mean	Standard Deviation
**HL**	Access Reading Skills Comprehension Assessment Decision-Making and Behavior	0-100	50.19 49.31 47.18 46.13 48.28	9.82 9.67 9.74 9.32 9.59
	**Total HL**	**0-100**	**48.22** ^*^	**9.63**
**QOL**	Physical Health Psychological Health Social Relations Living Environment	7–35 6–30 3–15 8–40	19.41 12.95 9.01 20.22	5.53 4.87 3.25 5.61
	**Total QOL**		**61.59** ^**^	**12.43**

* Score out of 100

** Score out of 120

The findings revealed a positive and significant correlation between HL and its dimensions with QOL among older people (*r*=0.388, *p*<0.001). Among the HL dimensions, comprehension had the highest correlation with QOL (*r*=0.411, *p*<0.001) (Table 3).

**Table 3:** Correlation between older people’ HL and QOL

		HL						Total HL
**QOL**	**Dimension**		Access	Reading Skills	Comprehension	Assessment	Decision-Making and Behavior	
	Physical Health		*r*=0.442 *p*<0.001	*r*=0.455 *p*=0.001	*r*=0.492 *p*<0.001	*r*=0.429 *p*=0.001	*r*=0.475 *p*<0.001	*r*=0.457 *p*<0.001
	Psychological Health		*r*=0.459 *p*<0.001	*r*=0.444 *p*=0.001	*r*=0.506 *p*<0.001	*r*=0.431 *p*=0.002	*r*=0.453 *p*<0.001	*r*=0.486 *p*<0.001
	Social Relations		*r*=0.383 *p*=0.001	*r*=0.371 *p*=0.002	*r*=0.394 *p*<0.001	*r*=0.351 *p*=0.003	*r*=0.389 *p*<0.001	*r*=0.379 *p*=0.001
	Living Environment		*r*=0.259 *p*=0.002	*r*=0.253 *p*=0.002	*r*=0.282 *p*=0.001	*r*=0.221 *p*=0.004	*r*=0.271 *p*=0.001	*r*=0.247 *p*=0.003
**Total QOL**			*r*=0.384 *p*<0.001	*r*=0.372 *p*=0.001	*r*=0.411 *p*<0.001	*r*=0.369 *p*=0.002	*r*=0.395 *p*<0.001	***r*** **=** **0.388** ***p***_**<**_ **0.001**

To determine the relationship of different HL dimensions with the QOL of older people, the results of the multiple linear regression analyses showed that the significant variables in the model determined using the Enter method were comprehension, decision-making and behavior, access, reading skill, and assessment, respectively. Moreover, the coefficient of determination in the processed model (R-Adjusted) of this test was 0.62, indicating that 62% of the variation in the QOL score can be explained by the variables in this model. According to the multiple linear regression analyses, the linear equation of the participants’ QOL was obtained as follows:

$$\begin{array}{l}Y = 1.813{\rm{ }} + {\rm{ }}0.461\,{x_1} + {\rm{ }}0.434\,{x_2}\\\,\,\, + {\rm{ }}0.397\,{x_3} + {\rm{ }}0.362\,{x_4} + {\rm{ }}0.278\,{x_5}\end{array}$$

Where, *Y* is the QOL score, and *x* represents different dimensions of HL in the studied population (Table 4).

**Table 4:** Multiple linear regression test results for variables affecting older people’s QOL

Variables		Unstandardized coefficients		Standardized coefficient *β*	t-statistics	P-Value
		*β*	Std. Error			
**---**	(Constant)	1.813	0.312	-	3.21	0.001
**x**_**1**_	Comprehension	0.461	0.099	0.384	2.87	<0.001
**x**_**2**_	Decision-Making And Behavior	0.434	0.084	0.371	2.65	<0.001
**x**_**3**_	Access	0.397	0.076	0.339	2.57	<0.001
**x**_**4**_	Reading Skill	0.362	0.065	0.296	2.49	0.002
**x**_**5**_	Assessment	0.278	0.053	0.228	2.08	0.004

The results showed significant differences among participants’ mean HL scores regarding gender (*p*=0.04) and level of education (*p*=0.001). In this regard, the mean HL scores of older people women (48.58 ± 9.74 out of 100) and those with bachelor’s degrees and higher education (49.48 ± 10.22 out of 100) were higher. Moreover, there were significant differences among the mean scores of older people’s QOL regarding their gender (*p*=0.02), marital status (*p*=0.03), level of education (*p*=0.002), and income (*p*=0.01). Accordingly, the mean QOL scores were higher for the females (62.55 ± 12.56 out of 120) than the males, for the married older people (64.97 ± 13.28 out of 120), for those with bachelor’s degrees and higher education (64.42 ± 13.16 out of 120), and for the participants with the income level above 30 million Rials per month (65.25 ± 13.48 out of 120) (Table 5).

**Table 5:** Relationship between HL and QOL regarding participants’ demographic information

Variables	Category	HL		QOL	
		Mean ± SD (From 100)	P-Value	Mean ± SD (From 120)	P-Value
**Age (year)**	60–70	49.53 ± 10.26	0.10	63.94 ± 13.22	0.08
	71–80	48.52 ± 9.63		61.17 ± 12.47	
	80<	46.61 ± 8.67		59.66 ± 12.17	
**Gender**	Male	47.86 ± 9.32	**0.04**	60.63 ± 11.86	**0.02**
	Female	48.58 ± 9.74		62.55 ± 12.56	
**Marital Status**	Single	48.83 ± 9.49	0.09	63.49 ± 12.61	**0.03**
	Married	49.07 ± 9.83		64.97 ± 13.28	
	Divorced	46.56 ± 9.61		59.21 ± 11.29	
	Widow	48.42 ± 9.55		58.69 ± 11.76	
**Level of Education**	Unable to Read and Write	47.31 ± 8.36	**0.001**	58.71 ± 11.53	**0.002**
	Reading and Writing	47.82 ± 8.62		59.19 ± 11.47	
	Elementary School	48.22 ± 9.35		62.05 ± 13.52	
	Diploma	48.27 ± 10.08		63.58 ± 12.37	
	BSc and higher	49.48 ± 10.22		64.42 ± 13.16	
**Income Level (Rials)**	< 10000000	46.21 ± 9.73	0.11	57.46 ± 11.93	**0.01**
	10000000–20000000	47.52 ± 9.62		59.31 ± 11.52	
	20000001–30000000	49.39 ± 10.11		64.34 ± 12.74	
	> 30000000	49.77 ± 9.82		65.25 ± 13.48	

## Discussion

The findings indicated the inadequate HL of older people. Consistent with this finding, Słońska et al. in Poland reported that most of older people aged 65 years and above had inadequate HL [60]. Moreover, in studies in different regions of Iran ( e.g., Kooshyar et al. [28], Ansari et al. [40], Borji et al. [43], Mohseni et al. [44], Reisi et al. [61]), most of older people’s HL level is reported to be inadequate. In comparison, Nezafati et al. [45] and TamizKar et al. [62] reported that more than half of older people had adequate HL. Similarly, Meier et al. in Switzerland found that 68.6% of older people had adequate HL [41]. According to Sørensen et al., HL adequacy in eight European countries ranged from 29 to 62% [63]. This inconsistency seems to be associated with the participants’ level of education in different studies. On the other hand, HL is a complicated construct, and several factors such as economic and social factors, cultural status, individual characteristics, and others may have effects on this construct. The results of the present study indicated the participants’ moderate QOL. Older people’s QOL has been reported differently in different studies. Similarly, Azadi et al. [47] and Moghadam et al. [64] reported moderate QOL for older people. According to a systematic review and meta-analysis, the QOL of the Iranian older people was almost moderate [65]. While Izadi et al. showed that the overall QOL of older people was at an acceptable level [66]. Miranda et al. in Brazil [67] and Rantakokko et al. in the United States [68] found out that most of older people had acceptable QOL. In addition to differences in the individual, social, economic, and cultural characteristics of the studied groups, the differences in the preparedness of different communities to face the challenges caused by aging can also be a reason for the inconsistencies in older people’s QOL in different societies.

The findings of the study revealed a statistically significant and positive correlation between older people’s HL and their QOL. Furthermore, the HL dimensions, including comprehension, decision-making and behavior, access, reading skills, and assessment, were significant relationship with QOL in older people. In Wang et al.’s study in China, high HL was significantly correlated with QOL in patients with high blood pressure [33]. Lee et al. showed that HL was one of the factors affecting health-related QOL in older people aged above 65 years in South Korea [39]. In the studies in Iran, there was a significant relationship between QOL and HL in older people [28, 69, 70]. Some studies have also documented that older people with a higher level of HL exhibit higher self-care behaviors and are healthier [46, 71–73]. Individuals with a higher level of HL may pay more attention to their health status and thus choose healthy behavioral habits, increasing their health-related QOL.

An investigation of the relationship between the main research variables and the participants’ demographic characteristics suggested that the participants’ mean HL scores were significantly different regarding their gender and level of education. Accordingly, older people women had higher HL than older people men. Moreover, with an increase in the level of education, older people’s HL increased as the HL of older people with a bachelor’s degree or higher education was higher than others. According to the results, the mean score of HL of the women was higher than that of men in Quaidi et al.’s study on patients aged above 40 years with type 2 diabetes [42]. In Ansari et al.’s study, the level of HL was higher in older people women [40]. Furthermore, Meier et al. showed that older people Swiss women’s HL was higher than that of men [41]. Perhaps, women’s search for more health-related content and more frequent visits to doctors and health centers to participate in screening programs and receive information from health workers are some reasons explaining their higher HL. Moreover, the role of women as the caretakers of other family members may encourage them to search for health-related information. Contrary to the results of the present study, older people men in some studies had higher HL [43, 74, 75]. The differences in individuals’ characteristics may be one of the reasons for inconsistencies in different studies.

In line with the results of this study, various studies have reported a significant relationship between HL and level of education; hence, the level of HL increases with an increase in individuals’ level of education [40, 41, 43–46, 74]. The elderlies with higher education have more ability to search for health-related content in virtual space and have more access to scientific information and texts. They can also establish effective communication with health care workers, and this has an effect on promoting their HL. In contrast to the aforementioned studies, Qaidi et al. provided different results in their study. In their study, the HL of all older people individuals with type 2 diabetes and a degree higher than a diploma was significantly lower than those with a diploma. The researchers concluded that a higher level of education does not guarantee higher HL [42]. Those with elementary education probably have more leisure time to search for health-related information from virtual social networks and receive information from mass communication media and have more opportunities to visit health centers for screening, care services, and information.

Finally, the mean score of older people’s QOL was significantly higher in older people women who were married and had a bachelor’s degree and higher education, with an income level above 30 million Rials. Accordingly, with an increase in older people’s level of education and income, their QOL increased.

In other studies in Iran (e.g., Azadi et al. [47], Rajabi et al. [48], Babak et al. [49], Raei et al. [50]), older people men’s mean QOL score was higher than older people women’s scores. Orfila et al. in Spain showed that older people women’s QOL scores were lower than that of men [76]. This contradicts the results of the present study. One of the reasons for older people men’s higher QOL scores in most studies may be the further financial independence of men than women. Moreover, in many cultures, after divorce or the death of a spouse, women have less chance of remarrying than men, and their isolation and loneliness may have a negative impact on their QOL. According to the results of the present study, the married older people had a higher QOL than older people who divorced from their spouses or whose spouses were not alive [44, 47, 48, 50–52]. One of the risks and problems of old age is loneliness and isolation; hence, attention to this issue and the provision of supportive and emotional conditions is one of the necessities of aging and affects the QOL of older people.

In line with the findings of this study, previous studies have claimed that the mean score of QOL increases with an increase in the level of literacy and education [47–49, 52, 53]. Given that higher education increases one’s competence in many fields, it can ultimately lead to higher QOL. The level of education not only is directly correlated with QOL by improving healthy behavior and lifestyle but also is indirectly by providing a better job, thereby making older people less vulnerable to economic and social problems and others. In line with the results of the present study, older people with higher incomes had a higher QOL [48, 50]. According to a review study in Iran, the results of studies on older people’s QOL revealed that this group is in a difficult situation due to the financial burden of the disease, and the researchers concluded that improving the economic situation can increase older people’s QOL [11]. Most elderlies may lose their source of income in old age and become economically dependent on others. On the other hand, medical expenses increase annually, and older people, especially those without additional insurance, are no longer able to pay medical costs. This implies that proper financial support is one of the factors affecting their QOL.

## Conclusion

The findings indicated inadequate and moderate levels of HL and QOL in older people, and there was a positive and significant statistical relationship between these two variables. In other words, improving HL leads to the improvement of QOL among older people. Inadequate HL in older people is considered a warning for officials, policy-makers, and health service providers, highlighting the need to pay further attention to HL in health promotion programs. Given that older people population in Iran is increasing, attention to the problems and needs of this group is a necessity. Accordingly, it is necessary to develop appropriate interventions to improve the HL level of older people to provide the grounds for improving their health status and promoting their QOL.

## Limitations

In this study, data collection was done by self-reporting, which may have an effect on the reporting of data by older people. Also, the use of a questionnaire and the inability to fully generalize the results to other societies and cultures are among the limitations of this study. Longitudinal studies in other regions with different cultures are highly recommended. As the study was conducted in Iran, it is recommended that similar studies be conducted in other countries so that a better understanding of the relationship between health literacy and quality of life can be obtained by comparing the results. However, despite the significant relationship between health literacy and quality of life in this study, there is a possibility that this relationship is influenced by a variety of factors, so future studies should explore the factors affecting the relationship between health literacy and quality of life.

## Data Availability

Whilst identifying/confidential patient data should not be published within the manuscript, the datasets used and/or analyzed during the current study are available from the corresponding author on reasonable request.

## Abbreviations

CVI: Content Validity Index
CVR: Content Validity Ratio
HELIA: Health Literacy for Iranian Adult
